# RNASEH2B loss and PARP inhibition in advanced prostate cancer

**DOI:** 10.1172/JCI178278

**Published:** 2024-06-04

**Authors:** Juliet Carmichael, Ines Figueiredo, Bora Gurel, Nick Beije, Wei Yuan, Jan Rekowski, George Seed, Suzanne Carreira, Claudia Bertan, Maria de Los Dolores Fenor de La Maza, Khobe Chandran, Antje Neeb, Jon Welti, Lewis Gallagher, Denisa Bogdan, Mateus Crespo, Ruth Riisnaes, Ana Ferreira, Susana Miranda, Jinqiu Lu, Michael M. Shen, Emma Hall, Nuria Porta, Daniel Westaby, Christina Guo, Rafael Grochot, Christopher J. Lord, Joaquin Mateo, Adam Sharp, Johann de Bono

**Affiliations:** 1 The Institute of Cancer Research, London, United Kingdom.; 2 The Royal Marsden NHS Foundation Trust, London, United Kingdom.; 3 Departments of Medicine, Genetics & Development, Urology, and Systems Biology, Columbia University Irving Medical Center, New York, New York, USA.

**Keywords:** Oncology, Prostate cancer

## Abstract

**BACKGROUND:**

Clinical trials have suggested antitumor activity from PARP inhibition beyond homologous recombination deficiency (HRD). *RNASEH2B* loss is unrelated to HRD and preclinically sensitizes to PARP inhibition. The current study reports on RNASEH2B protein loss in advanced prostate cancer and its association with RB1 protein loss, clinical outcome, and clonal dynamics during treatment with PARP inhibition in a prospective clinical trial.

**METHODS:**

Whole tumor biopsies from multiple cohorts of patients with advanced prostate cancer were interrogated using whole-exome sequencing (WES), RNA-Seq (bulk and single nucleus), and IHC for RNASEH2B and RB1. Biopsies from patients treated with olaparib in the TOPARP-A and TOPARP-B clinical trials were used to evaluate RNASEH2B clonal selection during olaparib treatment.

**RESULTS:**

Shallow codeletion of *RNASEH2B* and adjacent *RB1* — colocated at chromosome 13q14 — was common, deep codeletion infrequent, and gene loss associated with lower mRNA expression. In castration-resistant prostate cancer (CRPC) biopsies, RNASEH2B and RB1 mRNA expression correlated, but single nucleus RNA-Seq indicated discordant loss of expression. IHC studies showed that loss of the 2 proteins often occurred independently, arguably due to stochastic second allele loss. Pre- and posttreatment metastatic CRPC (mCRPC) biopsy studies from *BRCA1/2* WT tumors, treated on the TOPARP phase II trial, indicated that olaparib eradicated RNASEH2B-loss tumor subclones.

**CONCLUSION:**

PARP inhibition may benefit men suffering from mCRPC by eradicating tumor subclones with RNASEH2B loss.

**TRIAL REGISTRATION:**

Clinicaltrials.gov NCT01682772.

**FUNDING:**

AstraZeneca; Cancer Research UK; Medical Research Council; Cancer Research UK; Prostate Cancer UK; Movember Foundation; Prostate Cancer Foundation.

## Abstract 2

PARP inhibitor treatment clears subclones with RNASEH2B loss in metastatic castration-resistant prostate cancer, imparting clinical benefit in a subset of patients.

## Graphical abstract

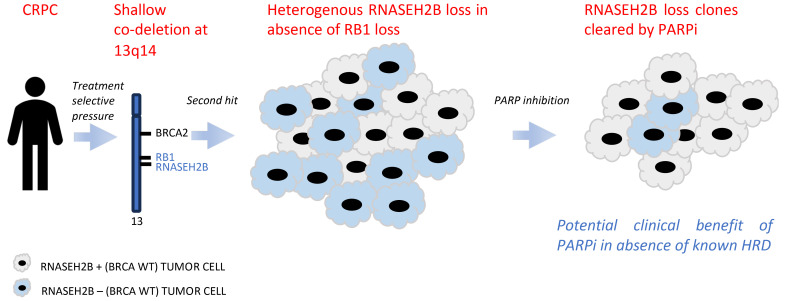

## Introduction

Prostate cancer (PC) is the second most common male malignancy worldwide, with over 1.4 million cases and 375,000 deaths per year (1). Progression to metastatic castration-resistant PC (mCRPC) after androgen deprivation therapy (ADT) is invariably fatal with a poor median overall survival (OS) of 2–3 years. Intra- and interpatient genomic heterogeneity are incontrovertible features of mCRPC, with 20%–30% of tumors harboring genomic aberrations related to DNA damage response (DDR), including *BRCA1/2* and *ATM* (2). DDR aberrations can sensitize to synthetic lethal therapies including poly (ADP-ribose)-polymerase inhibitors (PARPi) (3–5), with the PARPi olaparib transforming clinical practice by improving OS from mCRPC in patients with biallelic loss of *BRCA2* or *ATM* (6). Pronounced responses are mainly observed in the *BRCA2*-altered population, especially those with *BRCA2* homozygous deletion (7), but mixed responses are common in other molecular subgroups (8). Recent data combining androgen receptor signaling (ARSI) agents with PARPi for patients in molecularly unselected mCRPC suggest that PARPi may have broader antitumor activity beyond DDR-related gene alterations (9, 10). There remains an urgent need to validate predictive biomarkers identifying tumors sensitive to PARPi beyond *BRCA* gene alterations. Multiple preclinical screens have identified loss of function of RNASEH2B as being synthetic lethal with PARPi (11–13).

RNASEH2 is a heterotrimeric complex of 3 subunits (A-C), all key to its ability to remove misincorporated ribonucleotides from DNA by ribonucleotide excision repair (RER) (14). These lesions commonly arise during normal cellular processes including transcription (15), DNA replication (16), telomere elongation (17), and nonhomologous end joining (NHEJ) (18). Loss of RNASEH2 leads to an accumulation of misincorporated ribonucleotides and R loops in DNA, triggering DNA double-strand breaks (DSBs) (19), p53-mediated cell cycle arrest, and induction of DDR (20). Synthetic lethality between RNASEH2 gene loss and PARPi was identified using CRISPR screens (11, 12), with RNASEH2B loss sensitizing cells to PARPi in vitro to a similar extent as BRCA2 loss (13). Mechanistically, the absence of RNASEH2 permits alternative processing of ribonucleotide excision by topoisomerase 1, generating lethal PARP-trapping lesions that interfere with normal DNA metabolism by generating DSBs (11). Although loss of RNASEH2 function may occur in mCRPC, this remains inadequately investigated (11).

*RNASEH2B* is located on chromosome 13q. Large segments of chromosome 13q, including the *RB1* tumor suppressor, are commonly deleted in mCRPC, associating with poorer prognosis (21). *RB1* loss in PC is typically subclonal and can be detected at diagnosis before treatment, but loss increases at mCRPC with subclonal RB1 loss in 56% of mCRPC biopsies by fluorescence in-situ hybridization (FISH) in our previously reported studies (22). *RNASEH2B* is adjacent to *RB1* (within 2.5 Mb on 13q14.3), with whole-biopsy data indicating that the 2 genes may be codeleted. Studies suggest that RB1 protein coloss with RNASEH2B can decrease PARPi sensitivity (13), so studying RNASEH2B also needs to consider RB1 coloss. We hypothesized that subclonal RNASEH2B protein loss emerges at mCRPC due to treatment selective pressure resulting in RB1 loss. The current study characterizes RNASEH2B protein loss in mCRPC, its association with RB1 protein loss, its impact on clinical outcomes, and its relevance to treatment with PARPi in a prospective clinical trial.

## Results

### Chromosome 13 shallow deletions encompassing RB1 and RNASEH2B are common in mCRPC and decrease RB1 and RNASEH2B mRNA transcripts.

**Figure 1:**
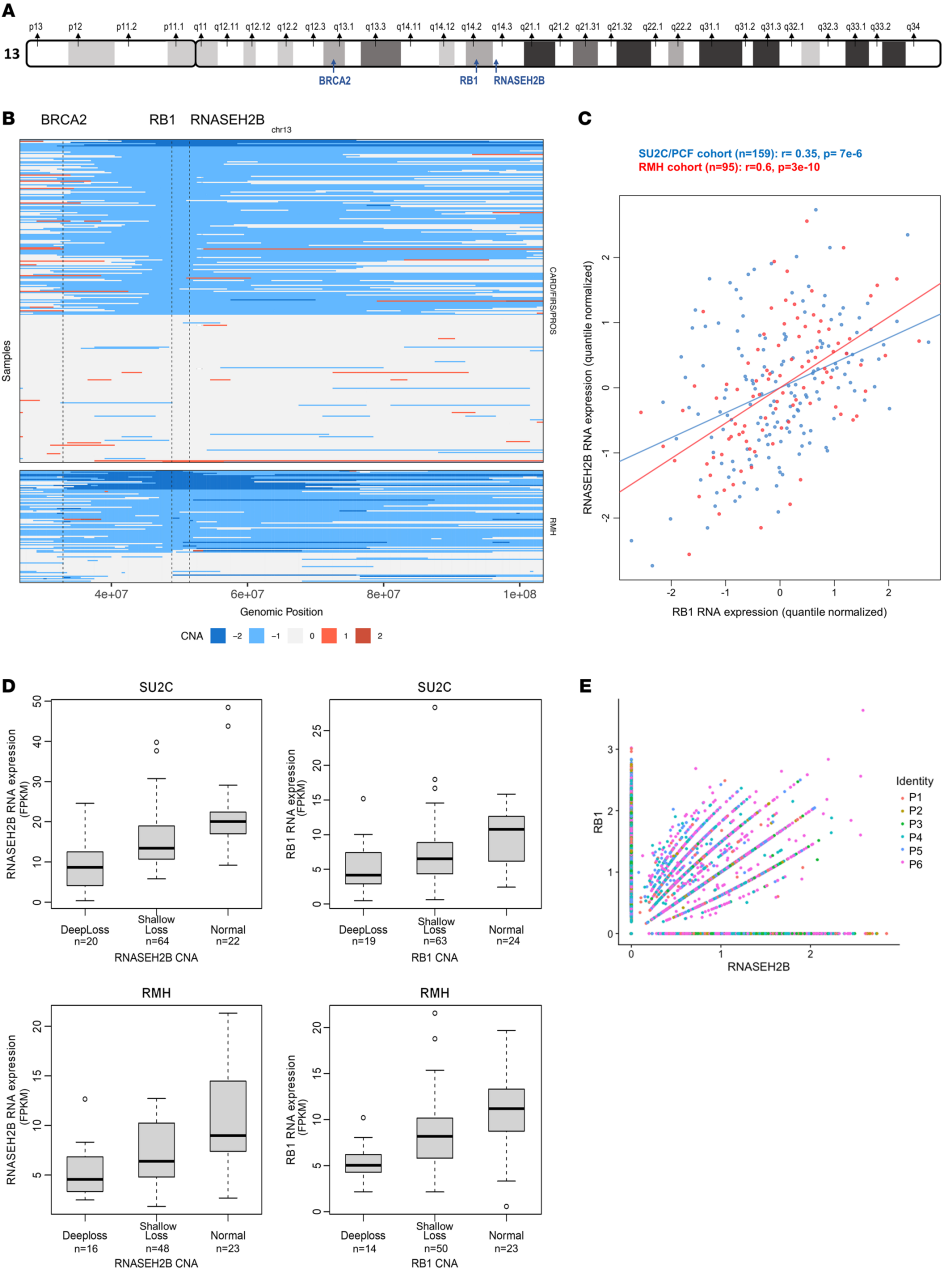
RNASEH2B and RB1 gene expression in CRPC. (**A**) *RNASEH2B*, *RB1* and *BRCA2* are located in close proximity on chromosome 13. (**B**) *RNASEH2B* and RB1 deletions, most frequently shallow, were commonly observed in whole mCRPC biopsies from a RMH whole-exome cohort (*n =* 93) and lpWGS of plasma DNA from 267 patients treated in 3 clinical trials (FIRSTANA, PROSELICA, and CARD). (**C**) Scatter plot of *RNASEH2B* and *RB1* mRNA expression (quantile normalized) in the SU2C/PCF (blue) and RMH (red) CRPC cohorts. *r* and *P* values were calculated using Spearman correlation. (**D**) Association between copy number and RNA expression of *RB1* and *RNASEH2B* in the SU2C/PCF (*n =* 106) and RMH cohorts (*n =* 87), suggesting that, especially for the latter stage RMH cohort, detectable whole biopsy shallow loss at a DNA level is associated with loss of *RNASEH2B* expression. Horizontal bars denote IQRs and medians. Combined CNA and RNA expression was only present for a subset of the cohorts as depicted in **C**. (**E**) snRNA-Seq of 6 patients with CRPC demonstrating the expression of the RB1 and RNASEH2B gene in a single nucleus. lpWGS, low-pass whole genome sequencing; CNA, copy number alteration; IQR, interquartile range; CRPC, castration resistant prostate cancer; snRNA-Seq, single nucleus RNA-Seq.

*RNASEH2B* and *RB1* are adjacently located on chromosome 13q, along with *BRCA2* (Figure 1A). To investigate *RNASEH2B* and *RB1* genomic loss, chromosome 13 deletions were evaluated in circulating tumor DNA (ctDNA) low-pass whole genome sequencing (lpWGS) from patients with mCRPC before treatment with taxanes (*n =* 267), demonstrating that shallow deletions encompassing both *RB1* and *RNASEH2B* are common (present in 52% of the samples), and sometimes involve *BRCA2*, with deep deletions occurring infrequently (2% of the samples, Figure 1B). As ctDNA copy number alteration (CNA) analyses can be influenced by low tumor fraction, whole-exome sequencing (WES) of whole mCRPC tumor biopsies (*n =* 93) was also interrogated and demonstrated a similar pattern of *RNASEH2B* and *RB1* deletion, with shallow deletion occurring in 55% of samples and deep deletion in 18% of samples (Figure 1B). *RNASEH2B* and *RB1* mRNA expression were correlated in 2 separate CRPC cohorts (SU2C/PCF cohort, r = 0.35, *P =* 7 × 10^–6^; RMH cohort. *r* = 0.6, *P =* 3 × 10^–10^; Figure 1C); *RNASEH2B* and *RB1* mRNA expression decreased with increasing copy number loss (Figure 1D). Single nucleus RNA-Seq (snRNA-Seq) studies from 6 patients with mCRPC (*n =* 45,599 single epithelial nuclei) suggested that many nuclei had discordant loss of *RNASEH2B* and *RB1* mRNA (Figure 1E). Overall, these data suggested that frequent shallow genomic coloss of *RNASEH2B* and *RB1* occur in mCRPC. Subsequently, the question was raised how these results translated at a protein level.

### Validation of a RNASEH2B antibody for IHC.

**Figure 2:**
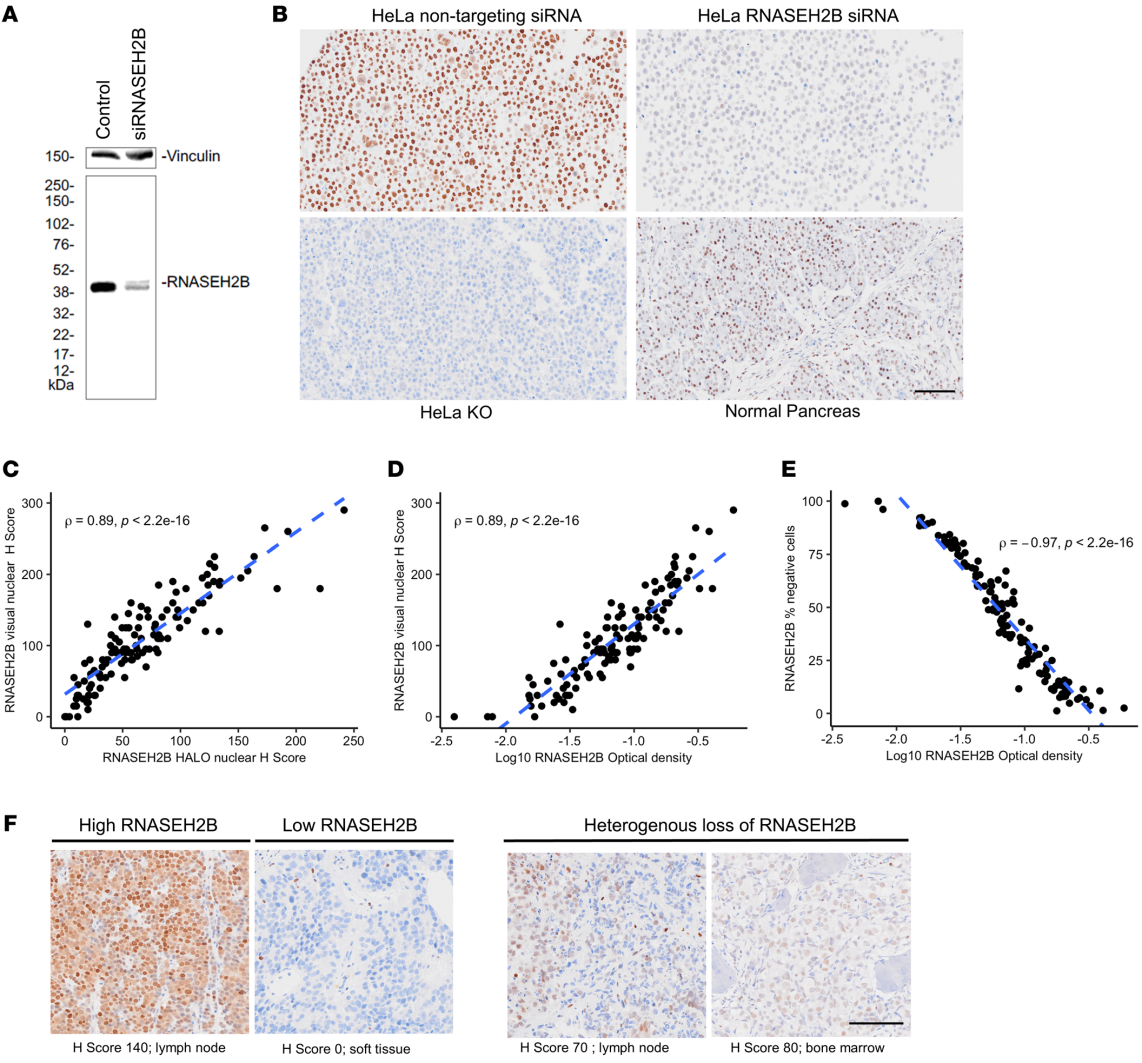
Validation and optimization of a RNASEH2B (RM433) antibody for IHC. (**A**) RNASEH2B antibody specificity confirmed by Western blotting of whole-cell lysates from HeLa cells treated with nontargeting control siRNA and pooled RNASEH2B siRNA. (**B**) IHC was run on HeLa cell pellets being treated with nontargeting control siRNA and pooled RNASEH2B siRNA, as well as HeLa RNASEH2B gene knock-outs and normal human pancreatic tissue. IHC depicted; magnification, × 10; scale bar: 100 μm. (**C**–**E**) Scatter plots showing associations between RNASEH2B IHC quantification by visual nuclear H score conducted by blinded pathologist and AI-trained HALO-generated OD, % negative cells and digital nuclear H Score. *r* and *P* values were calculated using Spearman correlation (**F**) Representative micrographs of RNASEH2B detection by IHC. Examples of high, low heterogenous (interspersed and sub-clonal) protein expression are shown. IHC depicted here; magnification × 10; scale bar: 100 μm. IHC, Immunohistochemistry; KO, knock-out; PC, prostate cancer; AI, artificial intelligence; OD, optical density.

To be able to evaluate RNASEH2B expression at a protein level, a RNASEH2B antibody was validated for IHC utilizing targeted RNASEH2B siRNA on both Western blot and a cell line pellet. Western blotting confirmed a single band corresponding with RNASEH2B expression in HeLa cell lysates treated with nontargeting control siRNA, which was reduced in lysates from HeLA cells treated with RNASEH2B-targeting siRNA (Figure 2A). Specificity was further confirmed by IHC of HeLa cell pellets treated with RNASEH2B-targeting siRNA, nontargeting control siRNA, and HeLa RNASEH2B CRISPR-knockouts (Figure 2B). Automated colorimetric digital (HALO) and visual analyses of RNASEH2B IHC data were correlated (Figures 2, C–E). HALO data were therefore utilized for analyses (1 sample was excluded due to unsatisfactory segmentation). Expression of RNASEH2B was predominantly nuclear, consistent with its known mechanism of action; nuclear H score alone was therefore used for IHC analyses. Both homogenous and heterogeneous RNASEH2B protein loss were identified in mCRPC biopsies. Image analyses revealed no detectable morphological difference between RNASEH2B positive and negative cells, with these being dispersed throughout mCRPC biopsy samples (Figure 2F). Overall, these data indicated that we had generated arguably the first validated RNASEH2B IHC antibody and confirmed RNASH2B protein loss in PC biopsies.

### Nuclear RNASEH2B protein loss is heterogeneous and decreases at mCRPC.

**Figure 3:**
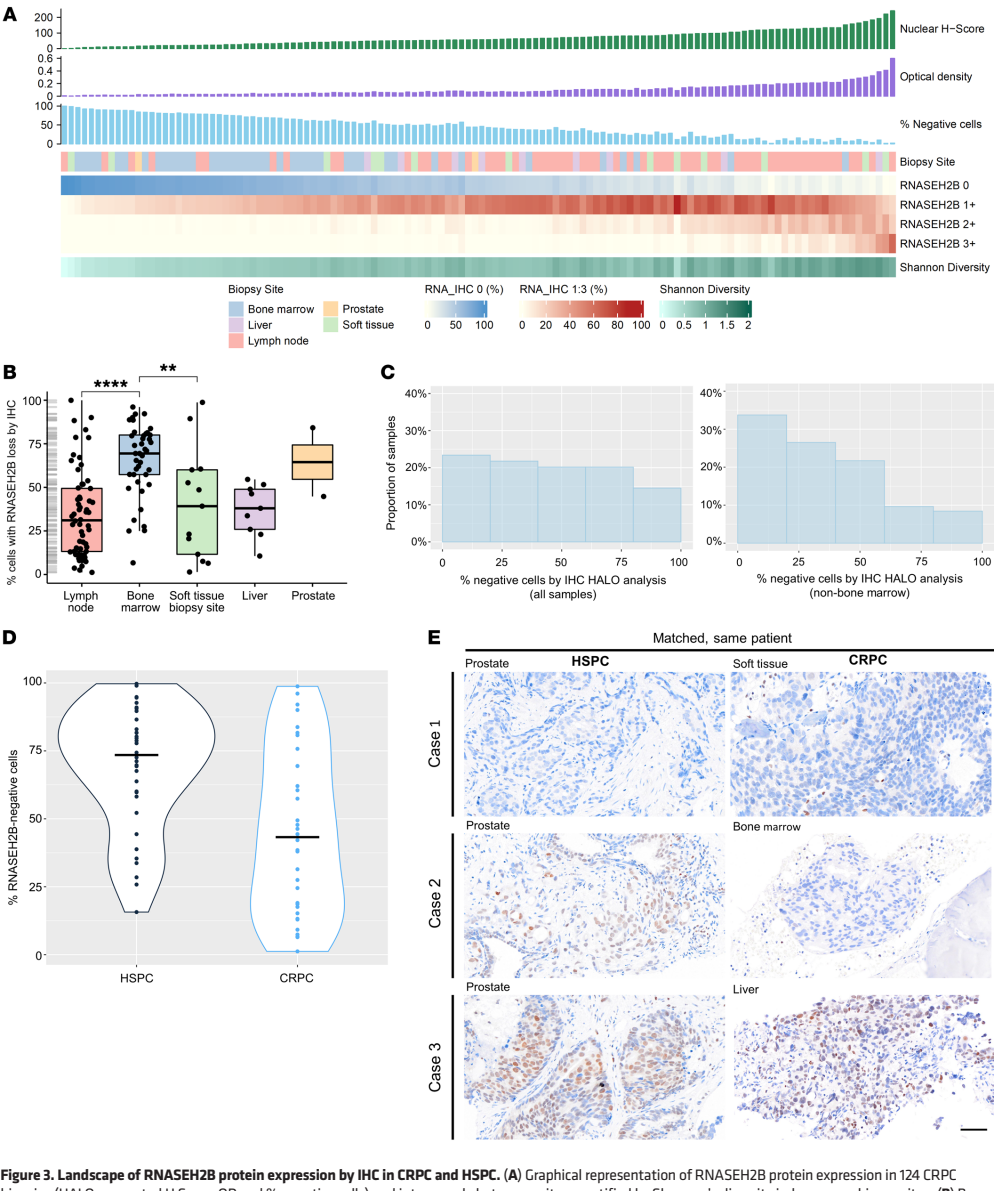
Landscape of RNASEH2B protein expression by IHC in CRPC and HSPC. (**A**) Graphical representation of RNASEH2B protein expression in 124 CRPC biopsies (HALO-generated H Score, OD and % negative cells) and intrasample heterogeneity, quantified by Shannon’s diversity index, across biopsy sites. (**B**) Box plot of RNASEH2B % loss by biopsy site, with plot to demonstrate the distribution. Horizontal bars denote IQR and medians. Kruskal-Wallis test was performed. (**C**) HALO was used to calculate the % RNASEH2B-negative cells by IHC in each sample, depicted as a histogram for all samples, and for nonbone marrow samples alone. (**D**) Violin plot of RNASEH2B-negative cells by IHC in paired, same-patient HSPC and CRPC biopsies (n = 34). Dots represent RNASEH2B-negative cells per sample, line represents median for whole group. (**E**) Representative micrographs of RNASEH2B detection by IHC in matched, same-patient HSPC and CRPC biopsies. Examples of complete RNASEH2B loss at HSPC and CRPC (case 1), and emergence of complete (case 2) or heterogeneous (case 3) RNASEH2B loss at CRPC are shown. IHC depicted here; magnification × 10; scale bar: 100 μm. IQR, interquartile range; CRPC, castration resistant prostate cancer; HSPC, hormone-sensitive prostate cancer; IHC, immunohistochemistry; OD, optical density.

RNASEH2B expression was evaluated by IHC in 124 CRPC biopsies from patients treated for CRPC at RMH in 2 different cohorts (cohort details in Supplemental Figure 1; supplemental material available online with this article; https://doi.org/10.1172/JCI178278DS1). Biopsies were taken from various metastatic sites, most commonly lymph nodes and bone marrow (Supplemental Table 1). Patients were generally pretreated with both an ARSI and taxane chemotherapy. Most patients had prostatic adenocarcinoma, while few (4/124, 3%) had neuroendocrine PC (NEPC). Marked intra-and inter-tumor heterogeneity in RNASEH2B expression were observed (Figure 3A). Most mCRPC biopsies revealed some tumor cell RNASEH2B IHC loss with 54 of 124 (44%) samples having loss in at least 50% of tumor cells, and 25 of 124 (20%) in at least 75% of tumor cells. Some mCRPC biopsies (11 of 124, 8.8%) had no RNASEH2B IHC staining. Negative RNASEH2B staining was consistent despite increasing concentrations of the primary RNASEH2B antibody (Supplemental Figure 2A). Overall, RNASEH2B IHC expression was lower in bone mCRPC biopsies, although loss was also observed in nonbone marrow samples (Figure 3, B and C). Therefore, bone decalcification protocols necessary for bone biopsy histopathology studies were tested on patient-derived mouse xenograft tissues to evaluate artifactual loss of staining (Supplemental Figure 2, B and C). The EDTA decalcifying agent did affect RNASEH2B staining and may have decreased RNASEH2B expression in bone biopsies, but RNASEH2B nuclear staining was usually still detectable despite this. In the 4 bone samples with more than 90% RNASEH2B-negative cells, stromal expression was observed (Supplemental Figure 2D), suggesting that loss of RNASEH2B was not entirely artifactual in these samples. Stromal protein staining may, at least in part, explain why mCRPC IHC staining quantitation did not correlate well with RNA expression data from a whole biopsy; this is denoted by 4 exemplar cases with complete loss of RNASEH2B on IHC (highlighted in red) that showed moderate-high levels of RNA expression in RNA-Seq data (Supplemental Figure 3A). RNA in situ hybridization (RNAish) for RNASEH2B confirmed this transcript’s more frequent loss in bone biopsies (Supplemental Figure 3B), with this correlating well with IHC (Supplemental Figure 3C), although it is possible that RNAish could also be impacted by decalcification.

RNASEH2B expression was also evaluated by IHC in matched, same-patient, hormone-sensitive PC (HSPC) and CRPC biopsies in 72 of the 125 (58%) patients where the HSPC sample was also available. A substantial number of HSPC samples failed quality control assessment (*n =* 37) due to weaker internal controls, and 1 sample did not have adequate tumor percentage. The number of RNASEH2B-negative cells appeared lower in CRPC (Figure 3D), but this analysis could be biased given the generally weaker internal controls in all HSPC samples, suggesting poor protein preservation. Exemplar micrographs of various RNASEH2B IHC expression from HSPC to CRPC are presented in Figure 3E. Overall, these data indicated that loss of nuclear RNASEH2B expression is common in CRPC and HSPC but is usually heterogeneous.

### RNASEH2B and RB1 proteins are differentially expressed.

**Figure 4:**
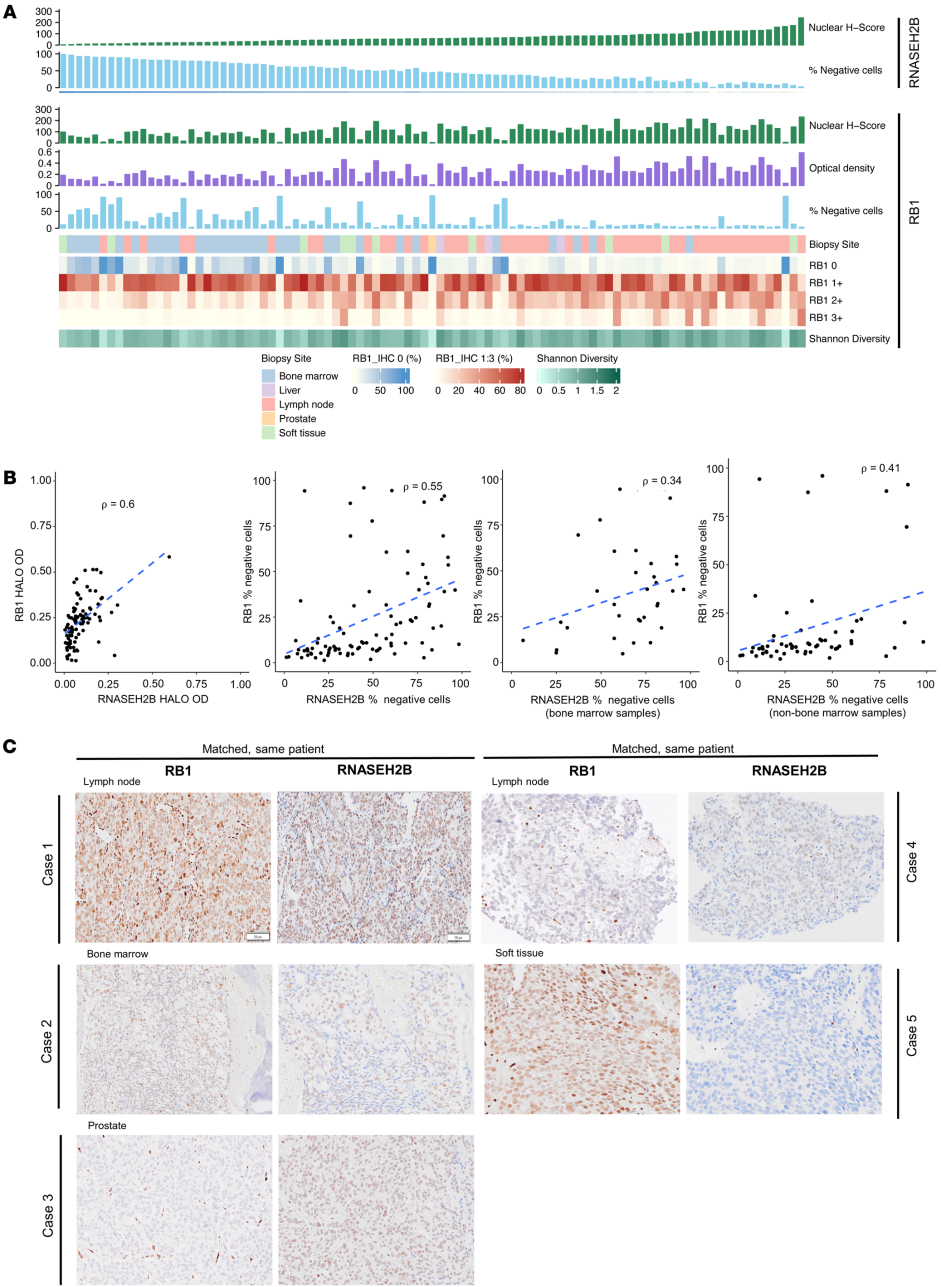
Evaluation of RB1 and RNASEH2B protein expression at CRPC by IHC. (**A**) Graphical representation of RB1 and RNASEH2B protein expression in 93 CRPC biopsies (HALO-generated H Score, OD and % negative cells) and intrasample heterogeneity, quantified by Shannon’s diversity index, across biopsy sites. Samples are matched, displayed in order of increasing RNASEH2B nuclear H score for both plots. (**B**) Scatter plot showing association between RNASEH2B and RB1 IHC quantification by HALO-generated % negative cells and OD. Scatterplots on the right distribute samples according to biopsy site, in bone marrow alone and nonbone marrow (soft tissue, liver, lymph node, prostate) samples. *r* and *P* values were calculated using Spearman correlation. (**C**) Representative micrographs of RB1 and RNASEH2B detection by IHC in matched, same-patient CRPC biopsies. Examples of concordant RNASEH2B and RB1 expression (case 1), heterogeneous loss of both RB1 and RNASEH2B (case 2), RB1 loss alone (case 3), RB1 loss with heterogeneous RNASEH2B (case 4) and RNASEH2B loss alone (case 5) at various biopsy sites are shown. IHC depicted here; magnification × 10; scale bar: 100 μm. While in a whole biopsy RB1 and RNASEH2B protein loss correlate, with both proteins being commonly heterogenously lost, surprisingly the data indicate that different cells in a biopsy often lose one protein or the other with only a minority of cells having coloss of both proteins. IHC, immunohistochemistry; IQR, interquartile range; CRPC, castration resistant prostate cancer; OD, optical density.

As sensitivity to PARPi in RNASEH2B-lost PC may be overridden by RB1 loss (13), RNASEH2B and RB1 protein coloss was investigated. An RB1 antibody (23) was validated. A single band corresponding to RB1 was observed in 22Rv1 cells, with marked reduction in RB1 detection in cells treated with RB1-targeted siRNA (Supplemental Figure 4A). This specificity was confirmed using IHC on 22Rv1 cell pellets treated with RB1 targeting or nontargeting control siRNA, and cells from the RB1-negative triple negative breast cancer (TNBC) cell line MDA-MB-468 (24) (Supplemental Figure 4B). Some background staining was observed, and this was accounted for in the HALO algorithm. As with RNASEH2B IHC, visual and digital (HALO) analyses correlated well (Supplemental Figure 4, C–E) and were utilized for the analyses. RB1 IHC was then performed on 93 of 125 (74%) of the CRPC biopsies with sufficient tissue. Surprisingly, RB1 protein loss was less frequent than RNASEH2B protein loss; 5 of 93 (5.4%) mCRPC biopsies had complete RB1 loss with many biopsies (over 60%) having a smaller proportion of cancer cells with RB1 loss (under 20% cells with RB1 loss), although heterogeneous loss of RB1 in mCRPC was also confirmed (Figure 4A). Interestingly, there were several cases with independent complete or heterogenous loss of 1 protein but not the other, with RNASHE2B loss being surprisingly more common than RB1 loss (Figure 4, A and B with exemplar micrographs in Figure 4C), and only 1 mCRPC biopsy had coloss of both proteins. Overall, these results indicate that the RB1 and RNASEH2B proteins are frequently independently lost at a cellular level, with coloss in the same cell being surprisingly less common; this would be in keeping with the hypothesis that stochastic but independent second allele loss occurs following shared heterozygous deletion of the chromosome 13 locus. This is supported by a general trend of positive correlation when investigating genes between RB1 and RNASEH2B using snRNA-Seq and bulk RNA-Seq from the SU2C cohort, in the absence of strong clustering among neighboring genes (Supplemental Figure 4, F and G).

### RNASEH2B loss is not an independent prognostic factor and does not associate with known signatures of DNA damage.

In keeping with this discordant loss of expression of RNASEH2B and RB1, there was no evidence for a significant association between median RNASE2H2B expression and established prognostic variables (Supplemental Figure 5, A and B). There was also no evidence for a significant overall difference in median RNASEH2B protein expression based on previous ARSI exposure (abiraterone or enzalutamide), or in relation to the time interval between CRPC diagnosis and CRPC biopsy (Supplemental Figure 5C).

The association between mCRPC RNASEH2B protein expression and OS did not appear to be linear; of note, patients with overall low or high RNASEH2B expression had a worse prognosis (Supplemental Figure 6A). Because of the absence of a linear relationship, nonlinear modelling was pursued with the univariate accelerated failure time (AFT) modelling revealing worse survival for patients with the highest RNASEH2B expression (Supplemental Figure 6B). However, in the multivariable model, once other prognostic factors were accounted for, minimal association between RNASEH2B expression and survival was observed (Supplemental Figure 6, C and D). RNASEH2B protein expression also was not correlated with the presence of other DDR aberrations including *BRCA2*, *PALB2*, *ATM*, *CDK12,* or MMR status (Supplemental Figure 7A). RNASEH2B IHC loss was also not significantly associated with established signatures of defective DDR, including telomeric allelic imbalance (NtAI) (25), large-scale transition (LST) (26) and homologous recombination defect loss of heterozygosity (HRD-LOH) scores (27), neither in the overall population or when excluding the impact of other DDR aberrations (Supplemental Figure 7B). These scores, which are increasingly used as a candidate predictive biomarker of PARPi response in other cancer types, would therefore not identify RNASEH2B-lost mCRPC.

### PARPi treatment impacts clonal selection of RNASEH2B-negative cells.

**Figure 5:**
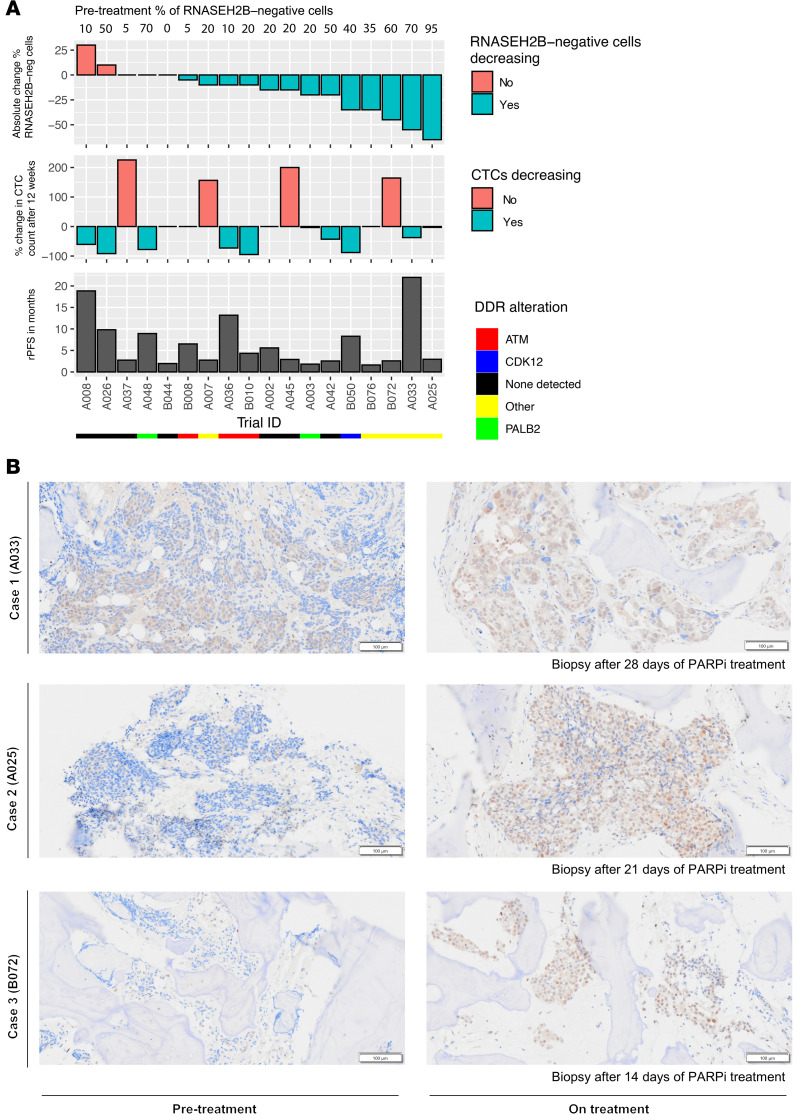
Changes in RNASEH2B expression in patients treated with PARP inhibitor olaparib in TOPARP trials. (**A**) Matched pretreatment and on-treatment biopsies were compared for RNASEH2B expression in patients without an identified *BRCA* alteration. Pretreatment percentage of RNASEH2B-negative cells are depicted above the waterfall plots. First waterfall plot depicts the absolute change in percentage of RNASEH2B-negative cells (on treatment % minus pretreatment %). Second waterfall plots depicts the percentage change in CTC number (by CellSearch) from pretreatment to 12-weeks of treatment. Tiles below depict which DDR alteration was identified in each specific patient. (**B**) Exemplar micrographs of RNASEH2B expression by IHC in the 3 cases with the largest percentage change in RNASEH2B-negative cells from pretreatment to on-treatment. IHC depicted here; magnification × 10; scale bar: 100 μm. CTC, circulating tumor cells; IHC, immunohistochemistry; DDR, DNA damage response.

Although preclinical data demonstrated a synthetic lethal relationship between RNASEH2B and PARPi, to date, evidence that this might operate in the clinic is lacking. To evaluate this, we assessed changes in RNASEH2B subclones following PARPi (olaparib) treatment in pretreatment and on-treatment samples from the TOPARP-A and TOPARP-B trials. Only patients without a *BRCA1/2* gene alteration were evaluated. The percentage of RNASEH2B-negative cells substantially decreased following PARPi treatment in most patients (13 of 18 patients) consistent with these cellular subclones being cleared by PARPi treatment (Figure 5A). We also observed decreasing CellSearch circulating tumor cell (CTC) counts on treatment in 6 of these patients, with 3 of these patients also having a relatively long rPFS despite the absence of *BRCA* gene loss (22 months in a patient with *FANCI* alteration; 13 months in a patient with *ATM* alteration; 8 months in a patient with *CDK12* alteration). Exemplar micrographs of the 3 patients with the largest changes in percentage of RNASEH2B-negative cells are depicted in Figure 5B. Together, these results suggest that RNASEH2B-negative tumor subclones are eradicated by PARPi.

## Discussion

RNASEH2B loss has been reported to be synthetic lethal with PARPi in multiple broad genetic perturbation screens (11–13). The current study characterized the landscape of RNASEH2B loss of protein expression in mCRPC. We demonstrate that the RNASEH2B protein is often lost heterogeneously and to varying extents in mCRPC subclones. Complete homogeneous RNASEH2B protein loss by IHC was uncommon and only detected in 8.8% of mCRPC biopsies, consistent with previously reported genomic data (12). Heterogeneous RNASEH2B loss was common with RNASEH2B lost in over 50% of cells in 44% of mCRPC biopsies (13). This loss was most common in bone biopsies and although this may have been partly attributable to bone decalcification, the presence of stromal RNASEH2B expression in the presence of tumor loss and similar RNAish data suggested this was not artifactual.

The current study builds on previous findings reporting on RB1 protein loss in CRPC, with this being usually heterogeneous (2, 28) and with shallow genomic loss being much more common than deep loss (29). We previously reported a comprehensive assessment of RB1 loss in matched HSPC/CRPC biopsies by whole genome sequencing (WGS), FISH, and IHC, and reported that RB1 loss increased at mCRPC, where 56% of patients had at least shallow RB1 deletion (22), which is in accordance with the IHC data presented in the current study. Surprisingly, despite RB1 and RNASEH2B correlating at a transcriptomic level, loss of RNASEH2B and RB1 protein expression by IHC was discordant at a cellular level. We hypothesize that this may be explained by monoallelic loss of RNASEH2B and RB1 occurring in the same cell, with the second hit occurring stochastically and less likely to occur in the same cell. Our finding that complete loss of both RB1 and RNASEH2B by IHC is uncommon is also in accordance with this hypothesis. The occurrence of a second hit is also supported by data from mCRPC biopsy genomics, where shallow loss of both is far more prevalent than deep loss of both. If RNASEH2B and RB1 loss of expression usually does not occur in the same cell, this may have clinical relevance given the recent observation that RB1 loss can limit PARPi sensitivity generated by RNASEH2B loss, perhaps through E2F1-mediated upregulation of homologous recombination repair (HRR) genes (13).

The extent of RNASEH2B loss required to sensitize to PARPi remains unknown, with studies primarily demonstrating sensitivity in CRISPR knockouts with complete loss of RNASEH2 function (11–13). One study reported double strand breaks, impaired NHEJ, and increased apoptotic cell death on small hairpin RNA depletion of both RNASEH2A and RNASEH2B in cell lines (30), suggesting that incomplete RNASEH2 loss can impact PARPi sensitivity, with at least one study in chronic lymphatic leukemia (CLL) models suggesting that monoallelic loss may sensitize cells to PARPi (11). Within patients with mCRPC treated with the PARPi olaparib in the TOPARP trials, we show herein that there are clonal dynamics within the RNASEH2B cell population. We report that RNASEH2B-negative subclones by IHC are cleared during PARPi treatment in most patients who are *BRCA*-WT, with this associating with evidence of clinical benefit in some individuals. The degree of benefit imparted is likely dependent on the proportion of tumor impacted by RNASEH2B loss as well as the molecular makeup of the tumor subclones that are not being cleared. This is supported by the observation that CTC counts decreased in individuals whose tumors had RNASEH2B loss, without any evidence of radiological benefit. These data suggest that clearance of RNASEH2B-loss clones may, at least in part, be responsible for the observed improved progression-free survival benefit with PARPi in some patients described as not having homologous recombination defects (HRD) in the PROPEL and TALAPRO-2 trials (9, 10). Importantly, we show that these patients cannot be identified using established DDR signatures and would thus be missed by these assays. Further studies are urgently required to validate these findings and extend the utility of PARPi beyond mCRPC with DDR defects, although this will not be easily feasible utilizing ctDNA studies and may require other biomarker analyses such as circulating tumor cell immunocytochemistry (31).

In summary, the data presented herein demonstrate that RNASEH2B loss of expression displays interpatient and intrapatient heterogeneity. At a single-cell level, RNASEH2B loss often occurs in the absence of RB1 loss, with RNASEH2B subclone loss being cleared by PARPi as previously indicated by multiple genomic screens. These data indicate that prospective studies of RNASEH2B loss need to be incorporated into PARPi predictive assays.

## Methods

### Sex as a biological variable.

Sex was not considered as a variable given the disease etiology.

### Patient and tissue samples.

Tissues from multiple cohorts were used for the analyses (Supplemental Figure 1). Main tissue analyses investigating RNASEH2B and RB1 IHC were performed with data from a single previously reported cohort (immune biomarker [IB] cohort (32)), and a not previously reported cohort of patients with mCRPC treated at the Royal Marsden Hospital (RMH), the RNASEH2B cohort. Eligible patients were required to have sufficient formalin-fixed, paraffin-embedded (FFPE) CRPC biopsy tissue. Tissues from the IB cohort were used for WES, targeted next-generation sequencing (NGS), and RNA-Seq. Patient-matched HSPC and CRPC biopsies for RNASEH2B IHC came from both the IB and the RNASEH2B cohort. Clinical and demographic data were retrospectively collected from electronic patient records.

For the correlative RNASEH2B and RB1 analyses, data from whole mCRPC biopsies with available WES from the IB cohort were analyzed to demonstrate CNAs as detailed before (32) at the locus of interest on chromosome 13. Chromosome 13 was also analyzed from lpWGS on ctDNA isolated from plasma samples from patients with CRPC treated within 3 previously reported clinical trials, FIRSTANA (33), PROSELICA (34), and CARD (35), using methods previously published (36). Shallow deletions were defined as a lpWGS log_2_ratio between –0.15 and –1; deep deletions were defined as a log_2_ratio less than –1. RNA-Seq and CNA data generated from the previously reported SU2C/PCF and RMH cohorts were analyzed as published before (22) to evaluate mRNA expression and CNA of *RNASEH2B* and *RB1*. For WES of bulk whole tumor biopsies, deep loss was defined as a CNA estimation equal to –2, and shallow loss or the cases with a CNA estimation equal to –1. For snRNA-Seq, single nuclei were acquired from 6 frozen mCRPC biopsies (4 lymph node, 2 liver metastases). Tissues for snRNA-Seq came from patients providing written informed consent as detailed above (reference 04/Q0801/60).

Tissues from patients participating in TOPARP-A or TOPARP-B (3, 4) were investigated for RNASEH2B RNAish and correlative analyses regarding the clearance of RNASEH2B sub-clones during treatment with olaparib.

### SnRNA-Seq.

Tumor biopsies were frozen in optimal cutting temperature compound (OCT) immediately after samples were acquired under ultrasound guidance. Single nuclei were obtained using a modified version of previously described methods (37). Briefly, after dissolving the OCT in cold 1 × PBS, tumors were dissociated by chopping the tissue for less than 5 minutes in cold TST lysis buffer (146 mM NaCl, 10 mM Tris-HCl pH 7.5, 1 mM Ca2Cl, 21 mM MgCl2, 0.05% Tween-20, 0.2 U/μl RNase inhibitor). Dissociated nuclei were first passed through a 70 μM filter and then a 40 μM filter, followed by centrifugation at 500*g* for 5 minutes at 4°C. The nuclei pellet was washed with NSB solution (1% BSA/PBS, 0.2 U/μl RNase inhibitor) and then centrifuged at 500*g* for 5 minutes at 4°C. The nuclei pellet was resuspended in NSB solution.

snRNA-Seq was performed using the 10 × Genomics (Pleasanton) Chromium Single Cell 5**′** Library & Gel Bead Kit at the Columbia University Human Immune Monitoring Core (HIMC). Manufacturers’ protocols were followed for the preparation of gene expression libraries and the subsequent sequencing on the Illumina NovaSeq 6000 Sequencing System. The sequenced reads were processed by *Cellranger count* (v7.0.0) for cell calling using the default parameters and supplying an indexed hg38 genome as a reference, generated with the *Cellranger mkref command*.

A total of 73,692 nuclei were sequenced and 56,789 high-quality nuclei were obtained after filtering outliers, using the *Scuttle* (v1.4.0) *quickPerCellQC* function, which removed cells possessing library size, feature counts, and mitochondrial RNA content that lay 3 absolute deviations from the median. The filtered data was processed with *Seurat* (v4.3.0) and underwent normalization, scaling, clustering, and dimensional reduction before cell type assignment with *SingleR* (v1.8.1) using the *Blueprint ENCODE* reference data set from the *Celldex* (v1.4.0) package.

### Antibody validation and IHC.

We commissioned an antibody against RNASEH2B from RevMab Biosciences (Burlingame) in a collaborative effort (clone RM433 no. 31-1321-00). Antibodies against RNASEH2B and RB1 were validated for specificity by Western blot, comparing detection of protein in whole cell lysates treated with nontargeting control siRNA or ON-TARGETplus pooled siRNA against the target protein (Supplemental Table 2). IHC for RNASEH2B was performed using rabbit anti-RNASEH2B antibody (RevMab; controls and conditions are outlined in Supplemental Table 3). Sections were counterstained with hematoxylin. Cytoplasmic and nuclear quantification for each sample was determined by a pathologist blinded to clinical/molecular data using H scores ([% negative staining × 0] + [% weak staining × 1] + [% moderate staining × 2] + [%strong staining × 3]), to determine the overall percentage of positivity across the entire stained samples, yielding a range from 0 to 300. The heterogeneity in RNASEH2B expression was quantified with the Shannon Diversity Index (SDI).

An antibody titration (1:400, 1:200, and 1:50) was performed on representative biopsies to validate results. To explore the impact of decalcification on the RNASEH2B staining in bone marrow, an EDTA decalcification protocol was applied to 22Rv1 xenografts prior to RNASEH2B staining. Xenografts were incubated with EDTA solution (decalcifying agent) for 48 hours at 37°C after fixation with neutral buffered formalin (NBF).

Due to the EnVision system used for the main paper analyses being discontinued at the time the TOPARP IHC analyses were done, RNASEH2B IHC for the TOPARP-A/B cohorts was done using a reoptimized assay with Bond Polymer Refine system (Leica Biosystems). The same anti-RNASHE2B monoclonal antibody was used (RevMab Biosciences). Briefly, antigen retrieval was performed for 30 minutes with Bond ER1 solution, anti-RNASEH2B antibody (1:250 dilution) incubated with tissue for 30 minutes, and the reaction visualized using Bond Polymer Refine system (Leica Biosystems). Pancreas tissue was used as a positive control. Cell pellets from HeLa cells treated with control and RNASEH2B siRNA were used to confirm specificity of the antibody for RNASEH2B. Rabbit IgGs were used as negative control.

IHC for RB1 was performed using a mouse anti-RB1 antibody (23) (Cell Signaling Technologies, clone 4H1, no. 9309; controls and conditions outlined in Supplemental Table 3). Sections were counterstained with hematoxylin. Nuclear quantification for each sample was determined by a pathologist using H scores, as detailed above.

### RNAish.

RNAish detection was performed on 3 μm sections derived from FFPE blocks, with probes for RNASEH2B and PPIB (housekeeping gene for internal control of mRNA quality) on a BOND RX platform (Leica Biosystems) according to manufacturer’s protocol (Supplemental Table 4).

### Slide digitalization and artificial intelligence–assisted (AI-assisted) analysis.

Stained slides were scanned at high resolution using an Olympus Digital Slide Scanner (Slideview VS200) and analyzed using HALO software (Indica Labs). A supervised machine learning algorithm was trained to differentiate PC cells from stroma. The algorithm was optimized to provide optical density (OD) data for the intensity of nuclear staining in tumor and stroma for RNASEH2B and RB1. A threshold was defined to label cells as positive (strong/moderate/weak) or negative for each protein, producing the percentage of positive and negative cells in each sample and a HALO-generated H score. HALO and visual analyses correlated well, HALO being more accurate for RB1, as background staining was incorporated into the algorithm. HALO-generated H Score was therefore used for analyses, along with OD, and loss was defined as a HALO-generated H score of less than 15 after careful comparison between negative patient samples and HALO scores by a trained pathologist. To account for weaker RNASEH2B staining on HSPC biopsies, tumor cell OD was normalized to stromal OD for paired biopsies.

For RNAish, slides were scanned as above (40 × magnification) and analyzed using the RNAish analysis HALO module. Areas with PPIB expression less than 4 spots/cell were excluded and a threshold for positive and negative cells was defined.

### Western blotting.

Western blots were performed for antibody validation that were subsequently used for IHC (antibody details listed in Supplemental Table 3). Cells were lysed in RIPA buffer supplemented with PhosStop and protease inhibitors (1 tablet/10 mL RIPA). Lysates were collected with a cell scraper and kept on ice for 30 minutes, followed by sonication (15 seconds) and centrifugation (15 minutes at 4°C). Protein concentration was measured by BCA protein assay kit (Thermo Fisher Scientific). Protein extracts (25 μg) were separated on 4–12% NuPAGE Bis-Tris gel (Invitrogen) by electrophoresis and transferred onto Immobilon-PTM PVDF membranes (0.45 μm, Millipore). Membranes were incubated with red ponceau and blocked in blocking buffer (5 × milk TBST/5 × BSA TBST) for 1 hour, then incubated in primary antibody overnight at 4°C. Membranes underwent 3 5-minute washes in TBS-T before incubating in secondary antibody for 1 hour at room temperature. 3 further TBS-T washes were performed before chemiluminescence was detected using Clarity ECL Western blot detection substrate and visualized on the Chemidoc Touch imaging system (Bio-Rad).

### Defining DNA damage repair gene aberrations and DDR signatures.

Targeted NGS was performed using DNA extracted from CRPC biopsies and germline DNA, according to published protocols (3, 38). Results were used to classify patients according to underlying DDR aberrations. HRD scores (LST, HRD-LOH, and NtAI) were calculated with HRDetect (39) using ASCAT (40) output from exome sequencing analysis and correlated with RNASEH2B protein expression.

### Statistics.

Spearman’s rank-order coefficient was used to assess correlation. Differences in RNASEH2B expression across biopsy sites were evaluated with the Kruskal-Wallis test. OS from CRPC biopsy was defined from the date of mCRPC biopsy until the time of death, with patients still alive censored at date of last follow-up/contact (data freeze 19th July, 2022). RNASEH2B OD was used as a continuous variable to represent RNASEH2B expression. A Weibull distribution was assumed for OS to fit an AFT model studying the association between OS and log-transformed RNASEH2B OD assuming a linear relationship. As data was obtained from 2 separate patient cohorts, the model was adjusted for cohort. Restricted cubic splines with 3 knots were next used to allow modelling nonlinear relationships that account for shorter OS at the extremes of the log-transformed RNASEH2B OD scale. Linear and nonlinear models were initially run as univariate models. As the timing of the mCRPC biopsy was variable, reduced models adjusting for time from CRPC diagnosis to date of mCRPC biopsy and the patient cohort were run, followed by fully saturated multivariable models adjusting for known prognostic factors. χ^2^ test statistics for the multivariable analyses are presented. For the TOPARP analyses, response was defined in accordance with the primary analysis (3) as either: according to RECIST 1.1; a reduction in PSA of at least 50%, or a conversion in CellSearch CTC count (from ≥ 5/7.5 mL of blood to < 5). PSA and CTC changes were required to be confirmed at least 4 weeks later. Figures and graphs were generated using R v4.2.2.

### Study approval.

Analyses done in the IB, RNASEH2B and RMH internal cohort were done on samples from patients who provided written informed consent for institutional protocols approved through the RMH ethics review committee (reference 04/Q0801/60). Patients in the TOPARP studies provided written informed consent for institutional protocols approved through the London Surrey Borders ethics committee (REC reference 11/LO/2019).

### Data availability.

All data has been made available using the Supporting data values file as a part of the Supplemental Data. RMH and SU2C-PCF mCRPC cohort RNA-Seq and WES has been previously made available (2). All sequencing data are available through the European Genome-phenome Archive (https://ega-archive.org/) under accession EGAD50000000874. Further data access requests can be submitted to the corresponding author.

## Author contributions

JC was responsible for conceptualization, data curation, formal analysis, validation, investigation, visualization, methodology, project administration, funding acquisition, and writing and editing. IF was responsible for conceptualization, data curation, validation, investigation, visualization, methodology, and writing and editing. BG was responsible for conceptualization, data curation, formal analysis, investigation, visualization, methodology, and writing and editing. NB was responsible for data curation, investigation, and writing and editing. WY was responsible for software, investigation, formal analysis, methodology, data curation, supervision, and writing and editing. JR was responsible for software, formal analysis, investigation, visualization, methodology, and writing and editing. GS was responsible for data curation, software, formal analysis, and visualization. SC was responsible for data curation, Investigation, and software. CB was responsible for data curation, investigation, and software. MDLDFDM was responsible for data curation. KC was responsible for data curation. AN was responsible for validation. JW was responsible for validation. LG was responsible for software, formal analysis, and visualization. DB was responsible for software and visualization. MC was responsible for data curation. RR was responsible for data curation. AF was responsible for data curation. SM was responsible for data curation. JL was responsible for data curation. MMS was responsible for data curation. EH was responsible for data curation. NP was responsible for data curation and formal analysis. DW was responsible for data curation. CG was responsible for data curation. RG was responsible for data curation. CJL was responsible for formal analysis and writing and editing. JM was responsible for investigation and writing and editing. AS was responsible for supervision and writing and editing. JDB was responsible for conceptualization, resources, supervision, funding acquisition, project administration, and writing and editing.

## Supplementary Material

Supplemental data

ICMJE disclosure forms

Supporting data values

## Version 1 06/04/2024

In-Press Preview

## Version 2 11/01/2024

Electronic publication
