# Supplementary material for: RNASEH2B loss and PARP inhibition in advanced prostate cancer
Source: J Clin Invest. 2024 Jun 4;134(21):e178278. doi: 10.1172/JCI178278 (PMC11527451; doi:10.1172/JCI178278)
Supplement: Supplemental data [file jci-134-178278-s223.pdf]

SUPPLEMENTAL MATERIAL

Full conflict-of-interest statement for CJL:

CJL receives and/or has received research funding from AstraZeneca, Merck KGaA, and Artios and has received consultancy, SAB membership or honoraria payments from Syncona, Sun Pharma, Gerson Lehrman Group, Merck KGaA, Vertex, AstraZeneca, Tango, 3rd Rock, Ono Pharma, Artios, Abingworth, Tesselate, Dark Blue Therapeutics, Pontifax, Astex, Neophore, and Glaxo Smith Kline. CJL also has stock in Tango, Ovibio, Hysplex, Tesselate and is a named inventor on patents (see full list below) describing the use of DNA repair inhibitors and stands to gain from their development and use as part of the ICR "Rewards to Inventors" scheme and reports benefits from this scheme associated with patents for PARP inhibitors paid into CJL's personal account and research accounts at the Institute of Cancer Research.

Patent list for CJL:

WO2023203229 (A1) – 2023-10-26
WO2022122938 (A1) 2022-06-16
US2022387544 (A1) 2022-12-08
US2019008856 (A1) 2019-01-10
WO2018224536 (A1) 2018-12-13
ES2671233 (T3) 2018-06-05
DK3044221 (T3) 2018-04-23
ES2611504 (T3) 2017-05-09
WO2014013231 (A1) 2014-01-23
WO2013024282 (A2); WO2013024282 (A3) 2013-02-21
WO2008020180 (A2); WO2008020180 (A3) 2008-02-21
WO2009027650 (A1) 2009-03-05
US2011212101 (A1) 2011-09-01
WO2009063175 (A1) 2009-05-22
PCT/FR2024/000031

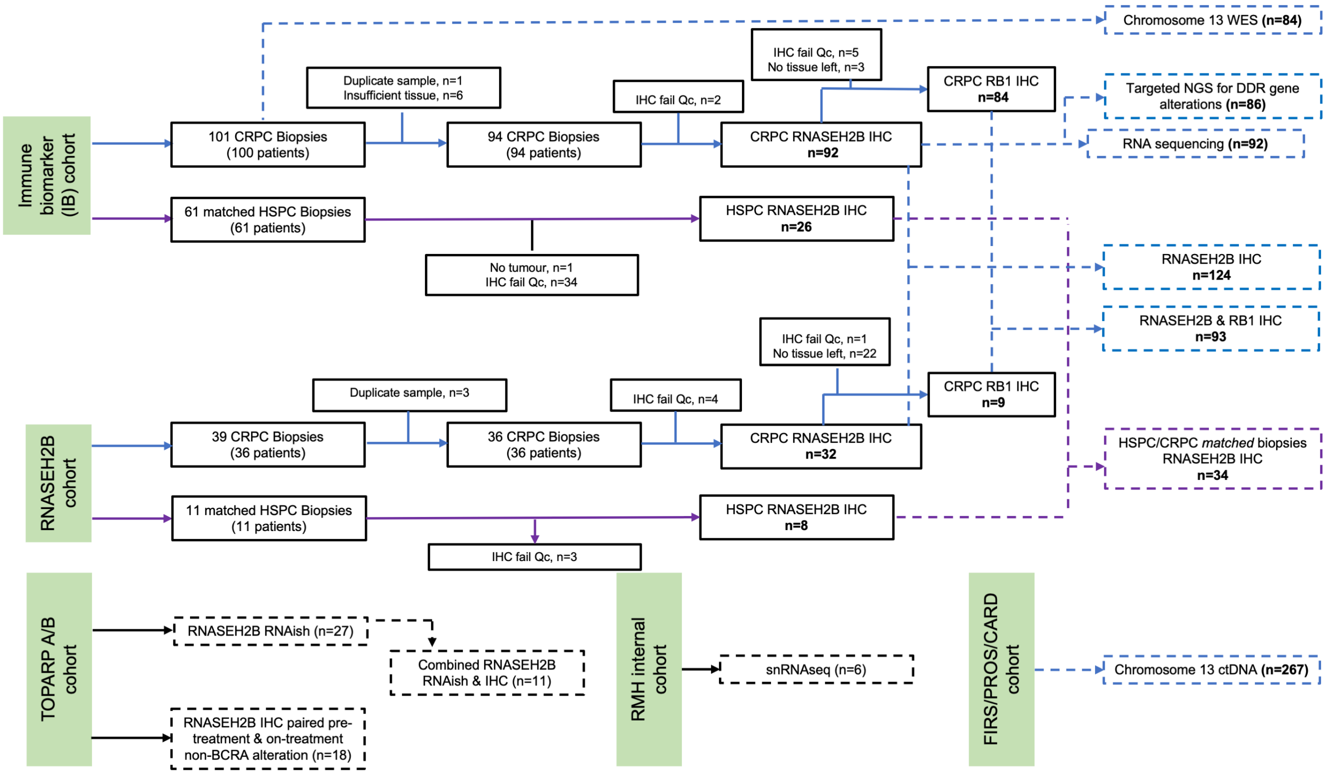

Figure S1. Summary of clinical samples analyzed Overview of the patient cohorts utilised for this study without publicly available data. The Immune biomarker (IB) patient cohort included 92 CRPC, of which 84 were also evaluated for RB1 expression, and 26 HSPC biopsies stained for RNASEH2B expression. Most of the patients in this cohort had NGS, WES and RNAseq data available. The RNASEH2B cohort included 32 CRPC and 8 HSPC biopsies stained for RNASEH2B expression, of which 9 were also evaluated for RB1 expression. In total 34 HSPC/CRPC matched same-patient biopsies were evaluated for RNASEH2B expression. The TOPARP-A/B cohort included patients with RNASEH2B RNAish (n=27) and matched RNASEH2B RNAish & IHC (n=11), with 18 non-*BRCA* altered patients being evaluable for changes in RNASEH2B expression at baseline and on-treatment whilst on olaparib. A group of mCRPC patients treated at the RMH, not fitting into any of the other cohorts, was evaluated using snRNAseq (n=6) to investigate RNASEH2B and RB1 expression. Lastly, the ctDNA of patients participating in FIRSTANA, PROSELICA and CARD phase 3 clinical trials was investigated to interrogate copy number alterations at chromosome 13. Clinical data was retrospectively collected for all patients. Interrupted lines represent final paper analyses. CRPC= castration resistant prostate cancer; ctDNA = circulating tumor DNA; HSPC= hormone-sensitive prostate cancer; IHC= Immunohistochemistry; Qc= quality control; NGS= next generation sequencing; WES= whole exome sequencing; RMH= Royal Marsden Hospital; RNAseq= RNA sequencing; RNAish=RNA in-situ hybridization; snRNAseq= single nucleus RNA sequencing

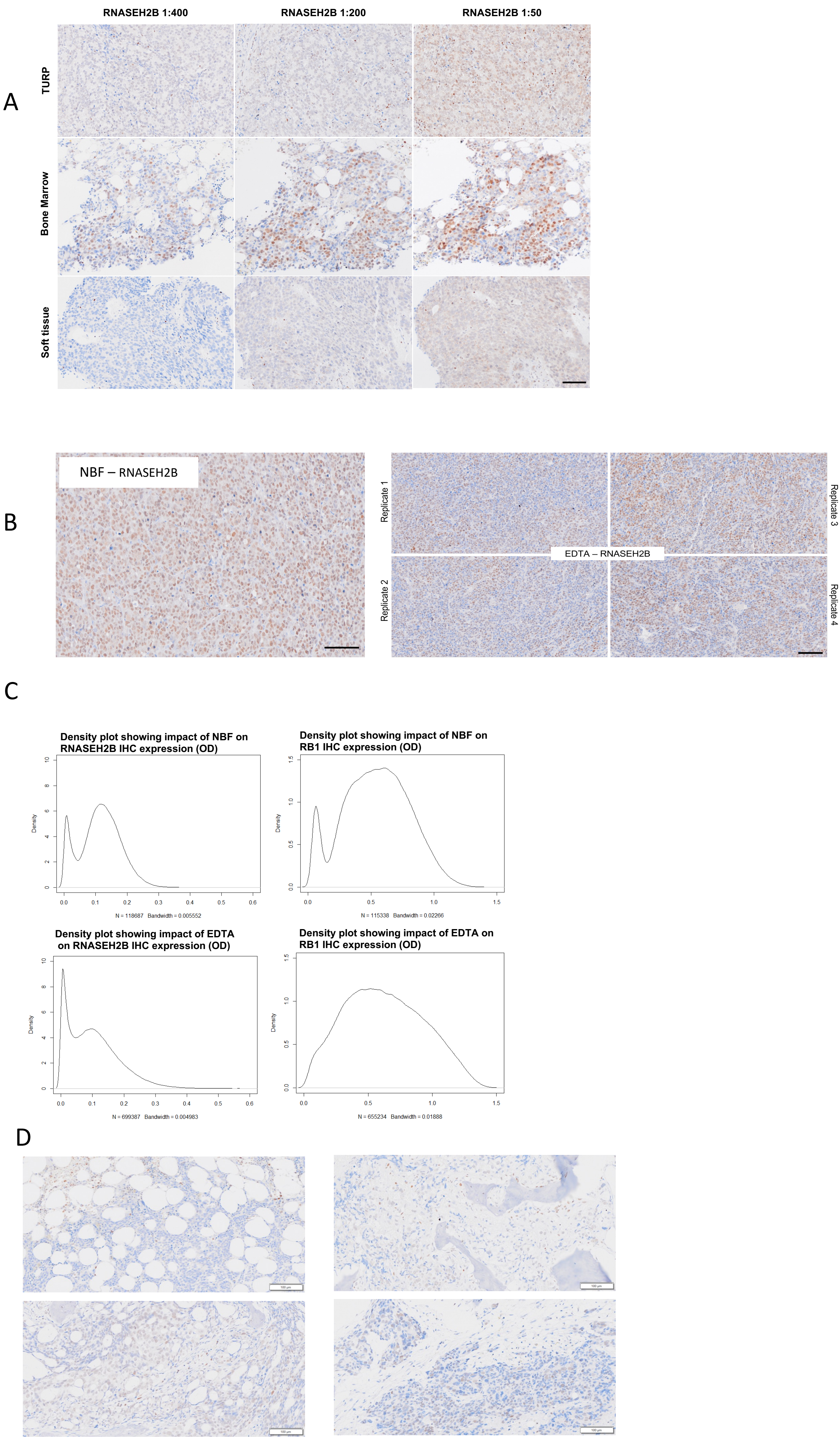

Figure S2: Validation experiments for RNASHE2B IHC assay (A) An antibody titration (1:400, 1:200, 1:50) was performed on biopsies from different sites, demonstrating that RNASEH2B-negative cells remained negative at higher antibody concentrations. **(B)** To assess the impact of decalcification on RNASEH2B assay in bone marrow, an EDTA decalcification protocol was applied to 22Rv1 xenografts prior to RNASEH2B staining and compared to NBF alone. Xenografts were incubated with EDTA solution (decalcifying agent) for 48 hours at 37°C) after fixation with NBF and demonstrated the decalcification protocol influenced RNASEH2B to some extent **(C)** HALO analysis showing impact of NBF and EDTA on RNASEH2B and RB1 IHC staining (nuclear OD). **(D)** Exemplar micrographs of four bone biopsies with >90% RNASEH2B-negative cells demonstrating stromal RNASEH2B-positive cells, suggesting that the high percentage of RNASEH2B-negative cells is not entirely artefactual. All IHC depicted here with x10 magnification and 100µm scale band. EDTA= Ethylenediaminetetraacetic acid**;** NBF= neutral buffered formalin.

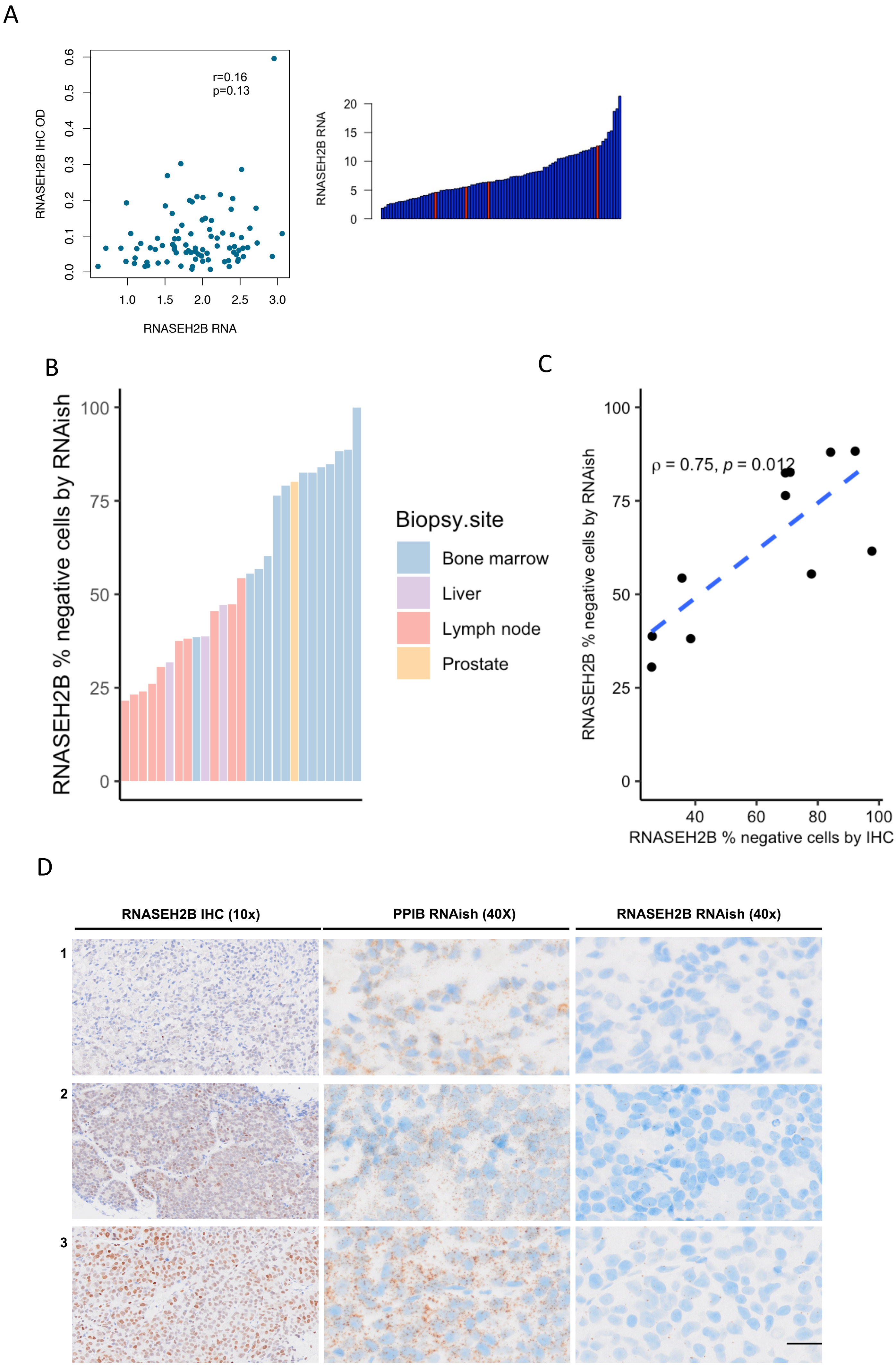

Figure S3: Correlation between RNASEH2B RNA expression (RNAseq, RNAish) and protein expression (IHC). (A) Scatterplot depicting correlation between whole biopsy RNASEH2B RNA expression (RNAseq) and RNASEH2B protein expression (OD) in the IB cohort (n=92). Barplot demonstrates distribution of RNA expression by RNAseq in 92 CRPC biopsies, with IHC negative cases highlighted in red, indicating that whole biopsy RNA analyses does not identify tumor biopsy RNASEH2B protein loss **(B)** RNAish was performed on 27 CRPC biopsies taken at baseline for patients on the TOPARP-B study with probes for RNASEH2B and PPIB, analyzed with HALO. Areas with PPIB expression less than 4 spots/cell were excluded and a threshold for positive and negative cells was defined. The percentage of PPIB-high tumor cells negative for RNASEH2B is depicted here in a barplot according to biopsy site. **(C)** Due to overlap between the two cohorts, 11 CRPC samples from 10 patients were stained for RNASEH2B protein expression by IHC and RNASEH2B RNA expression by RNAish. Scatterplot depicts correlation between % RNASEH2B negative cells by IHC and RNAish according to HALO analysis in 11 matched CRPC samples. *r* and *P* values were calculated using Spearman correlation. **(D)** Representative micrographs of RNASEH2B detection by IHC and RNAish, with PPIB RNAish shown to depict RNA quality. Examples of complete (1) and heterogeneous (2, 3) RNASEH2B loss are shown. IHC depicted here with x10 magnification and 100µm scale bar, RNAish with x40 magnification and 20µm scale bar. IHC= Immunohistochemistry; CRPC= castration resistant prostate cancer. OD= optical density.

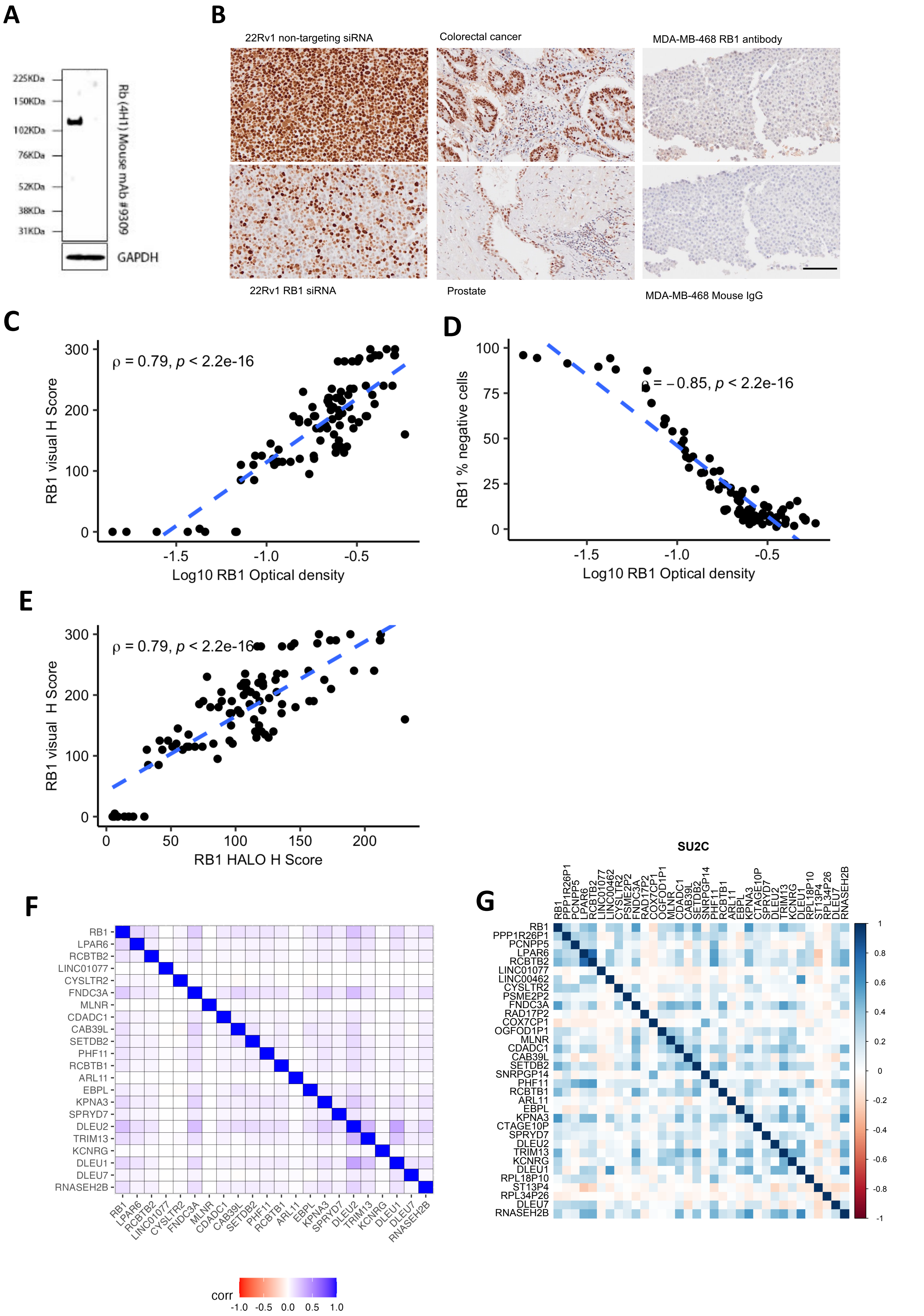

Figure S4: RB1 antibody validation and correlation of RB1-RNASEH2B neighboring genes (A) RB1 antibody specificity confirmed by western blotting of whole-cell lysates from 22Rv1 cells treated with non-targeting control siRNA and pooled RB1 siRNA. **(B)** In addition, IHC was run on 22Rv1 cell pellets treated with non-targeting control siRNA and pooled RB1 siRNA, as well as human benign prostate and colorectal cancer tissue. RB1 negative TNBC cell line, MDA-MB-468, was stained for RB1 and mouse IgG (negative control). IHC depicted here with x10 magnification and 100µm scale bar. **(C-E)** Scatter plots showing associations between RB1 IHC quantification by visual H score conducted by blinded pathologist and AI-trained. **(F)** Spearman correlation of neighboring genes of RB1 and RNASEH2B as determined using snRNAseq. **(G)** Spearman correlation of neighboring genes of RB1 and RNASEH2B as determined using bulk RNAseq from the SU2C cohort. IHC= Immunohistochemistry; AI= artificial intelligence; OD= optical density, TNBC=triple negative breast cancer.

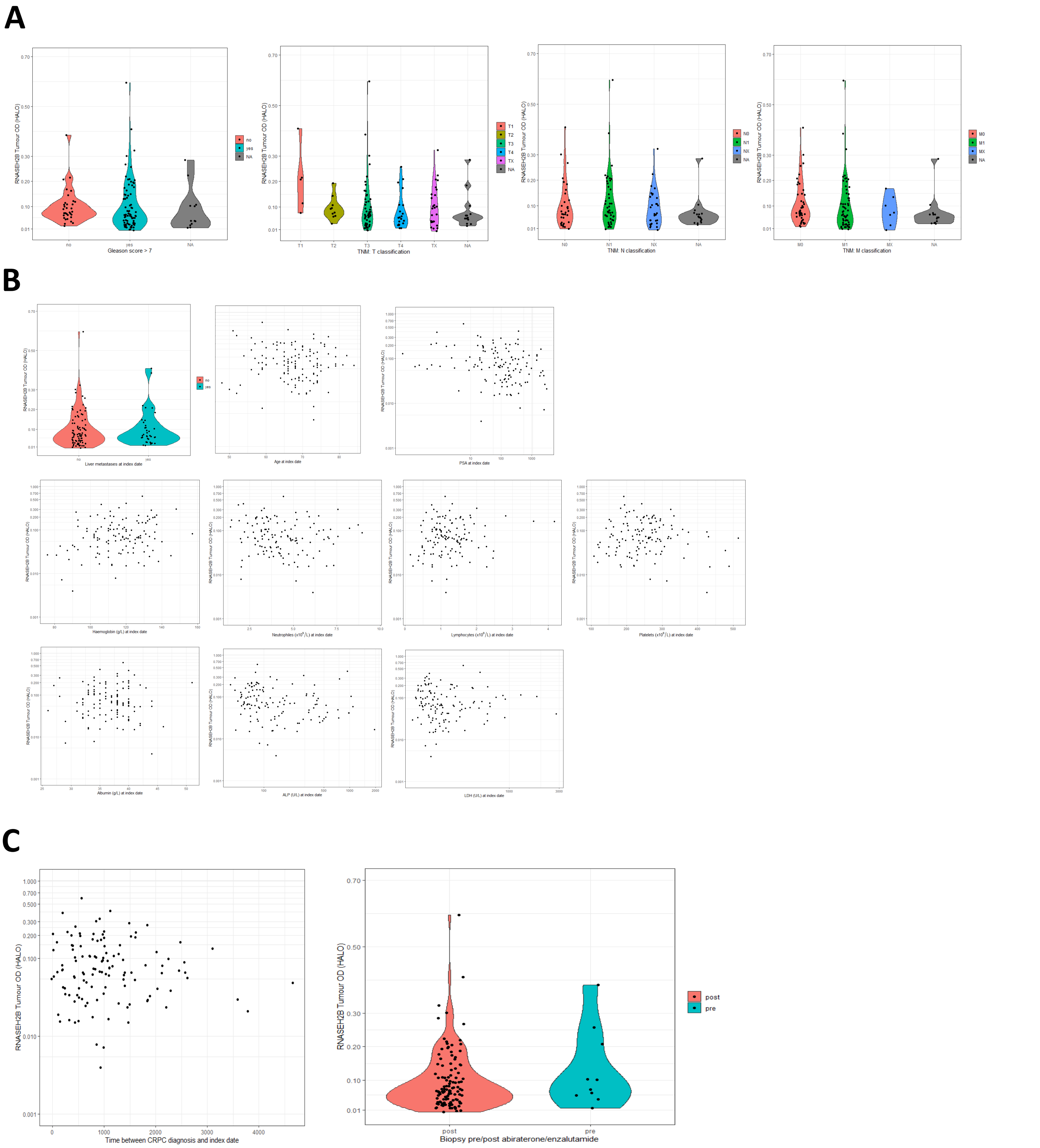

Figure S5: Association between RNASEH2B protein expression and CRPC prognostic indicators (A) Violin plots showing association between RNASEH2B OD and baseline prognostic indicators, including TNM stage and Gleason score. **(B)** Scatterplots and violin plot showing association between RNASEH2B OD and CRPC prognostic indicators. **(C)** Scatterplot showing association between RNASEH2B OD and time between CRPC diagnosis and CRPC biopsy; and violin plot showing RNASEH2B OD in patients who had or had not received an NHA, to ascertain whether RNASEH2B loss emerges due to treatment selection pressure by NHA treatment. OD= optical density; CRPC= castration-resistant prostate cancer; ARSI= androgen receptor signalling agent.

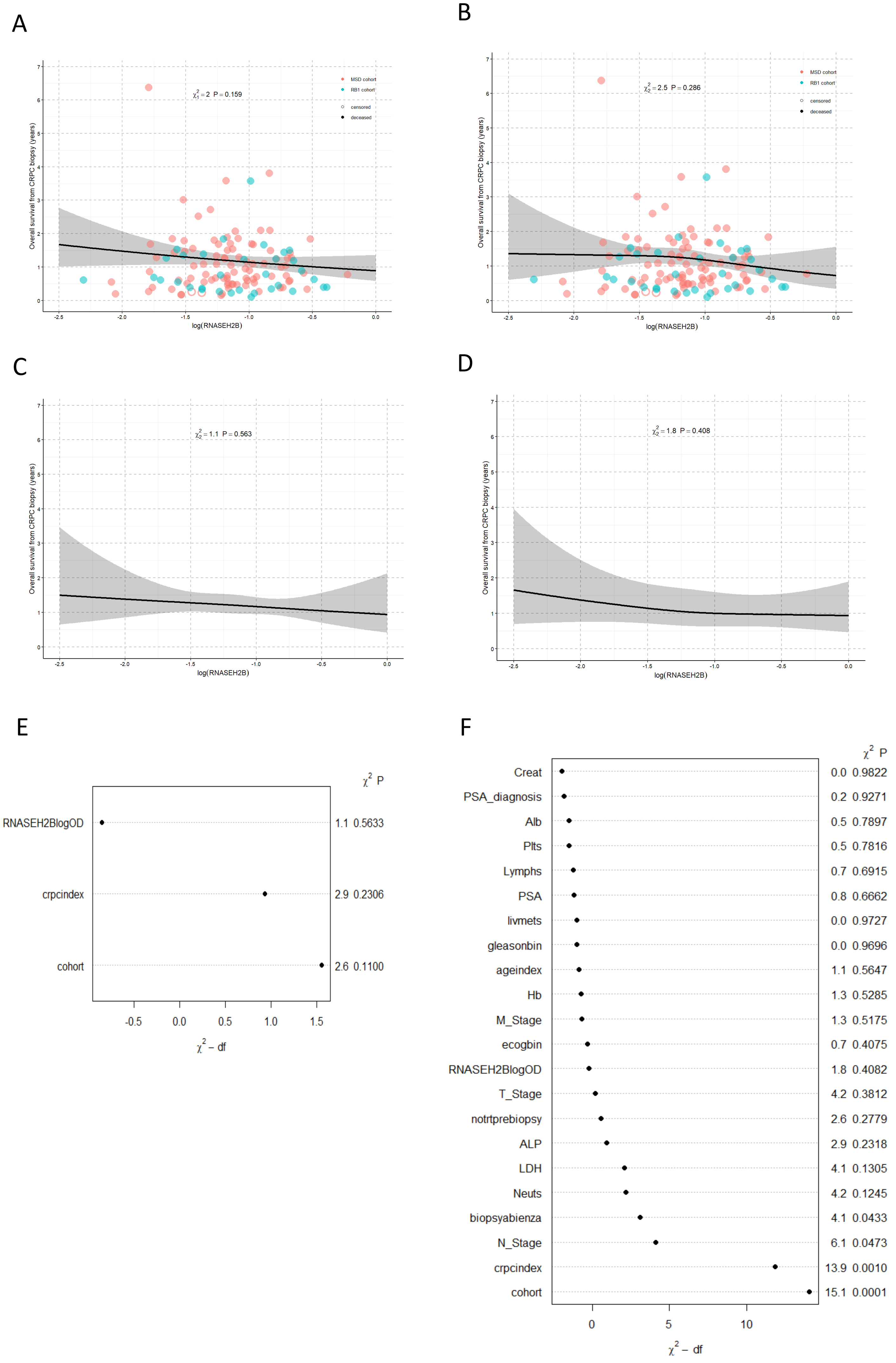

Figure S6: Impact of RNASEH2B protein expression on survival outcomes (A). Univariate linear AFT model showing OS from CRPC biopsy association with RNASEH2B expression at CRPC, measured as RNASEH2B OD by HALO analysis of IHC on 124 CRPC biopsies. **(B)** Univariate non-linear AFT model showing OS from CRPC biopsy according RNASEH2B expression at CRPC. **(C)** Reduced multivariate non-linear AFT model showing OS from CRPC biopsy according RNASEH2B expression at CRPC, accounting for time between CRPC diagnosis and CRPC biopsy and cohort only. **(D)** Saturated multivariate non-linear AFT model showing OS from CRPC biopsy according RNASEH2B expression at CRPC, accounting for all prognostic variables. Chi-squared test statistics with their respective p-values for all variables are shown **(E)** Result of multivariable AFT model including RNASEH2B OD, patient cohort (IB and RNASEH2B cohort) and time between CRPC and CRPC biopsy. **(F)** Variables included in saturated multivariate non-linear AFT model including only patients with all variables available (n=84). Chi-squared test statistics with their respective p-values for all variables are shown. AFT= accelerated failure time; CRPC = castration-resistant prostate cancer; IHC = immunohistochemistry; OS= overall survival; OD= optical density

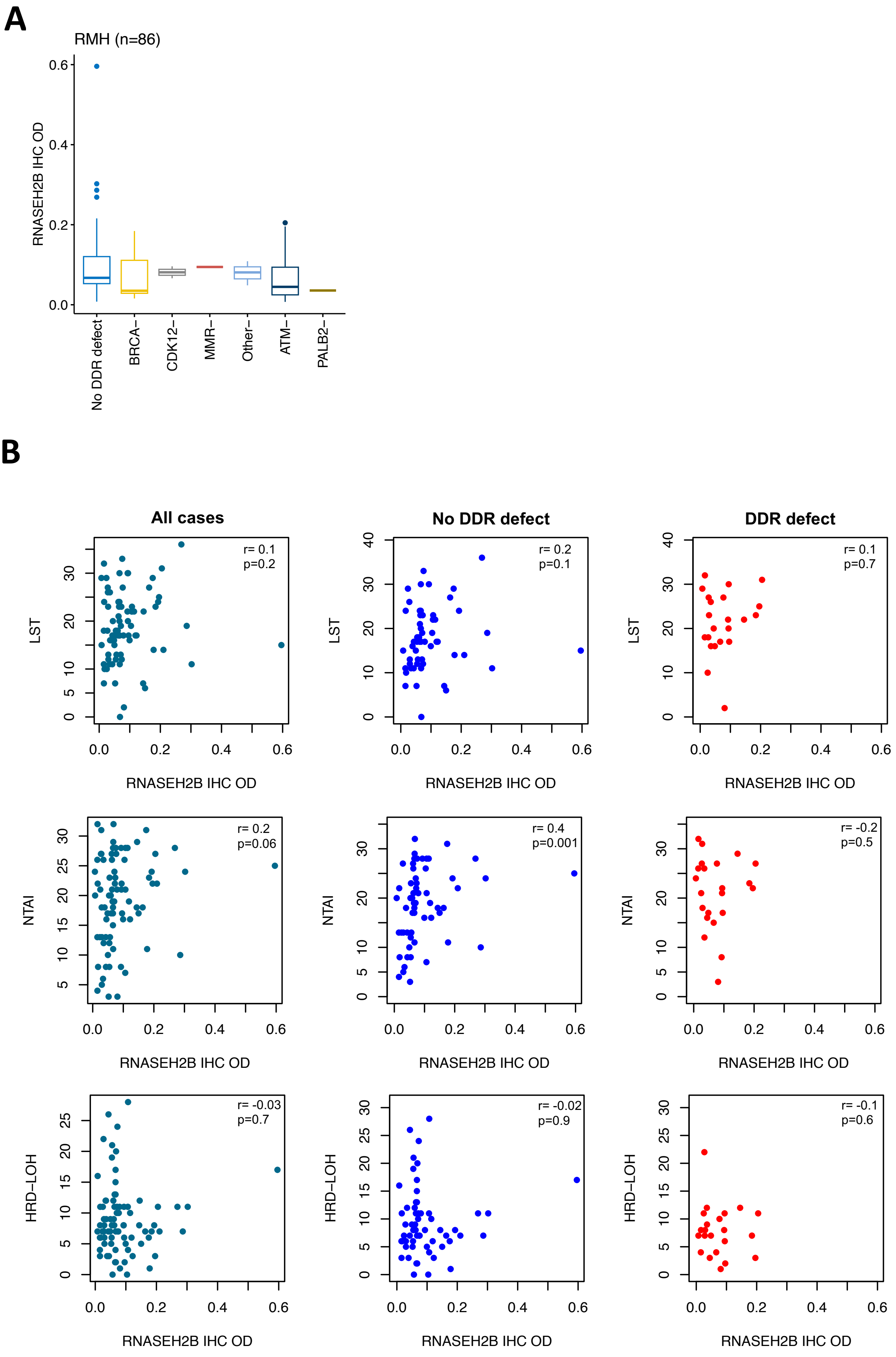

Figure S7: Association between RNASEH2B expression, deleterious DDR alterations and signatures of DNA damage (A) Targeted NGS data from CRPC biopsies from RNASEH2B cohort (n=86) was used to classify patients according to underlying DDR aberrations. IHC was used to quantify RNASEH2B protein expression, shown as OD according to HALO analysis. Boxplots depict the association between RNASEH2B protein expression and common DDR alterations in PC. Horizontal bars denote IQR and medians. **(B).** HRD scores (LST, HRD-LOH and NtAI) were calculated with HRDetect using ASCAT output from exome sequencing analysis and correlated with RNASEH2B protein expression, defined as HALO-generated OD. Scatterplots depict correlations, with additional plots separating plots according to DDR status. DDR= DNA damage response; NGS= next generation sequencing; PC= prostate cancer; OD= optical density; IQR= Interquartile range; HRD= Homologous repair deficiency; LST= Large scale transition; HRD-LOH= Homologous recombination deficiency- Loss of heterozygosity; NtAI= Number of Telomeric Allelic Imbalances; ASCAT= Allele-specific copy number analysis of tumors.

Supplementary Table S1: Patient characteristics

Patient characteristics	IB cohort (n=92)	RNASEH2B cohort (n=32)
**Age at diagnosis (years)**		
Median (IQR)	62 (58-67)	62 (57-64)
**CRPC histology, n (%)**		
Adenocarcinoma	92 (100)	28 (88)
Adenocarcinoma with NE differentiation	0	2 (6)
Small cell NE	0	2 (6)
**Gleason score at diagnosis, n (%)**		
10	2 (2)	0
9	46 (50)	14 (44)
8	11 (12)	6 (19)
7	24 (26)	7 (22)
≤6	2 (2)	0 (0)
NR	7 (8)	5 (15)
**Site of metastatic biopsy, n (%)**		
Lymph node	49 (53)	10 (31)
Bone	29 (32)	12 (38)
Liver	4 (4)	5 (16)
Soft tissue	9 (10)	4 (12)
Prostate	1 (1)	1 (3)
**Treatment prior to CRPC biopsy, n (%)**		
ADT	92 (100)	32 (100)
Abiraterone	57 (62)	21 (66)
Enzalutamide	45 (49)	10 (31)
Docetaxel	87 (95)	26 (81)
Cabazitaxel	43 (47)	8 (24)

IQR = interquartile range; CRPC= castration resistant prostate cancer; NE= neuroendocrine; NR= not recorded; ADT= androgen deprivation therapy.

Supplementary Table S2: ON-TARGETplus siRNA pools

siRNA Gene Target	Supplier	Catalogue No.
Control (non-targeting)	Dharmacon (Horizon)	D-001810-10
RNASEH2B	Dharmacon (Horizon)	L-014369-01
RB1	Dharmacon (Horizon)	L-003296-02

Supplementary Table S3: Antibodies and conditions for IHC

Protein	Supplier/ Catalogue No	Species	Instrument	Retrieval Method	Dilution/ Incubation Time	Detection System	Controls
RNASEH2B	RevMab Biosciences<br><br>#31-1321-00	Rabbit	BioGenex i6000 autostainer (Launch Diagnostics)	pH6 citrate buffer, pressure cooker	1:400<br><br>1 hour	Dako REAL EnVision Detection System (K4061, Agilent)	HeLa non-targeting siRNA /HeLa RNASEH2B siRNA<br><br>Positive control: Pancreas<br><br>Negative control: Rabbit IgG
			Bond RX (Leica Biosystems)	ER1 (AR9961, Leica Biosystems)<br><br>30 minutes	1:250<br><br>30 minutes	Polymer Refine Detection System (DS9800, Leica Biosystems)	
RB1	Cell Signaling Technology<br><br># 9309	Mouse	BioGenex i6000 autostainer (Launch Diagnostics)	pH6 citrate buffer, pressure cooker	1:1000<br><br>1 hour	Novolink Max Polymer DS (RE7280, Leica Biosystems)	22Rv-1 non-targeting siRNA/ 22Rv-1 RB1 siRNA<br><br>Positive control: Colorectal cancer and prostate<br><br>Negative control: Mouse IgG

Supplementary Table S4: Reagents for RNAish

Product	Supplier	Catalogue No.
RNASEH2B probe	Advanced Cell Diagnostics, CA	830418
Peptidylprolyl isomerase B (cyclophilin B - PPIB) probe	Advanced Cell Diagnostics, CA	313908
RNAscope 2.5 LS Reagent Kit-BROWN	Advanced Cell Diagnostics, CA	322100
